# A hybrid deep approach to recognizing student activity and monitoring health physique based on accelerometer data from smartphones

**DOI:** 10.1038/s41598-024-63934-8

**Published:** 2024-06-18

**Authors:** Lei Xiao, Kangrong Luo, Juntong Liu, Andia Foroughi

**Affiliations:** 1 Chengdu Technological University, Chengdu, 610000 China; 2 Graduate School of Business Faculty, Malaysia SEGi University, 47810 Petaling Jaya, Malaysia; 3 Chengdu University of Information Technology, Chengdu, 610000 China; 4 Department of Biomedical Engineering, Central Tehran Branch, Islamic Azad University, Tehran, Iran

**Keywords:** Human activity recognition, Student activity analysis, Sensor-based models, Edge computing, BiLSTM, Bayesian optimization, Health physique, Biomedical engineering, Health services

## Abstract

Smartphone sensors have gained considerable traction in Human Activity Recognition (HAR), drawing attention for their diverse applications. Accelerometer data monitoring holds promise in understanding students’ physical activities, fostering healthier lifestyles. This technology tracks exercise routines, sedentary behavior, and overall fitness levels, potentially encouraging better habits, preempting health issues, and bolstering students’ well-being. Traditionally, HAR involved analyzing signals linked to physical activities using handcrafted features. However, recent years have witnessed the integration of deep learning into HAR tasks, leveraging digital physiological signals from smartwatches and learning features automatically from raw sensory data. The Long Short-Term Memory (LSTM) network stands out as a potent algorithm for analyzing physiological signals, promising improved accuracy and scalability in automated signal analysis. In this article, we propose a feature analysis framework for recognizing student activity and monitoring health based on smartphone accelerometer data through an edge computing platform. Our objective is to boost HAR performance by accounting for the dynamic nature of human behavior. Nonetheless, the current LSTM network’s presetting of hidden units and initial learning rate relies on prior knowledge, potentially leading to suboptimal states. To counter this, we employ Bidirectional LSTM (BiLSTM), enhancing sequence processing models. Furthermore, Bayesian optimization aids in fine-tuning the BiLSTM model architecture. Through fivefold cross-validation on training and testing datasets, our model showcases a classification accuracy of 97.5% on the tested dataset. Moreover, edge computing offers real-time processing, reduced latency, enhanced privacy, bandwidth efficiency, offline capabilities, energy efficiency, personalization, and scalability. Extensive experimental results validate that our proposed approach surpasses state-of-the-art methodologies in recognizing human activities and monitoring health based on smartphone accelerometer data.

## Introduction

Human activity recognition (HAR) finds numerous practical applications, including monitoring individuals, detecting unusual or suspicious behavior, and overseeing patients in healthcare facilities^1^. HAR systems are extensively utilized across various domains such as behavior analysis^2^, gait analysis^3^, well-being management^4^, medical treatment^5^, urban areas leveraging advanced technology^6^, and daily activities^7^. HAR models are typically classified as vision-based or sensor-based depending on the type of information they process. These systems offer several advantages for research, including cost-effective installation, easy accessibility, user-friendliness, location independence, and energy efficiency. Common sensors like magnetometers, gyroscopes, and accelerometers are deployed in digital devices such as smartwatches and smartphones for collecting raw activity data^8^.

Some studies employ various models of traditional HAR, employing both machine learning and deep learning techniques. A fundamental aspect of HAR is the collection of sensor data, involving continuous data collection as participants engage in predetermined activities. Embedded sensors often encounter atypical data samples, which can be identified using outlier detection methods^9^. Subsequently, the data is segmented into equal intervals, followed by the extraction of distinctive features. Feature extraction requires careful consideration as it significantly impacts recognition efficacy. Primary domains for feature extraction include time–frequency, frequency, and time features. The categorization stage involves employing conventional Machine Learning (ML) classifiers to identify actions. Machine learning approaches are frequently preferred when there are sufficient labeled datasets, processing capacity, well-defined feature extraction techniques, and a short training period. Despite their advantages, ML classifiers have limitations such as the requirement for domain expertise, large quantities of unlabeled data, and the absence of a universal feature extraction method^10^. To overcome these challenges, Deep Learning (DL) methods are recommended. DL-based HAR comprises information collection, feature extraction, and model training and categorization stages^11^. Unlike machine learning approaches, deep learning models do not require data preprocessing, although specific preprocessing techniques can enhance outcomes. Deep learning models simultaneously train the model and extract features based on the quality of the raw dataset, determining the most valuable features and optimizing models accordingly.

The subject of student activity recognition and physical health monitoring holds significant importance due to its wide-ranging applications in educational settings, healthcare facilities, and personal well-being. Recognizing the significance of this topic, our paper aims to provide compelling explanations to attract researchers interested in these areas. Student activity recognition is crucial for educators and parents alike, as it offers valuable insights into students’ physical well-being and academic performance. By monitoring students’ activity levels, educators can identify patterns, trends, and areas of concern, allowing them to implement targeted interventions and support strategies to promote healthier habits and optimal learning outcomes. In addition, physical health monitoring plays a vital role in preventive healthcare, enabling early detection of potential health issues and promoting overall well-being. By utilizing accelerometer data from smartphones, our proposed approach offers a convenient and accessible means of tracking exercise routines, sedentary behavior, and overall fitness levels. This information can empower individuals to make informed decisions about their health and lifestyle choices, leading to improved quality of life and reduced healthcare costs.

In basic activity patterns, actions are executed linearly without complexity, overlap, or concurrency. DL methodologies have been integrated into various HAR applications due to their efficacy and utility. Researchers employ a range of deep learning architectures, including Long Short-Term Memory (LSTM), bidirectional LSTM (BiLSTM), recurrent neural networks (RNNs), and convolutional neural networks (CNNs), to understand spatial and temporal representations of sensor data. Restricting the proliferation of deep learning networks is advisable to achieve accurate activity detection while mitigating issues like overfitting, convergence difficulties, high memory requirements, and increased processing costs. Developing compact and lightweight networks that efficiently extract features remains a challenge. Further research is necessary to determine the optimal network structure for activity detection. Researchers are exploring small deep learning models to automate feature extraction from sensor data and enhance efficiency^12^. Focus on crucial components of sensor information is essential to optimize activity detection by eliminating extraneous or confusing information. Recognizing student activity and monitoring health via smartphone accelerometer data can contribute to effective education, promoting physical and mental well-being, and fostering an environment conducive to optimal learning outcomes. Our proposed feature analysis framework integrates smartphone accelerometer data with edge computing capabilities, allowing for real-time processing and analysis of vital signals directly on the device, thereby enhancing the efficiency and timeliness of human activity recognition and health monitoring. Furthermore, our utilization of Bidirectional LSTM (BiLSTM) and Bayesian optimization techniques addresses the limitations of traditional machine learning approaches by automating feature extraction and fine-tuning model architecture, leading to improved accuracy and scalability in HAR tasks. Based on the findings of this paper, the main contributions are as follows:

- Optimization of BiLSTM frameworks to accurately identify individuals and their behaviors, eliminating the need for manual feature extraction algorithms typical in traditional machine learning approaches. These models autonomously learn and extract valuable features from unprocessed sensory input.
- Evaluation of facial recognition performance using the publicly available UCI dataset revealed that the modified LSTM model outperformed the most advanced and recently published research in terms of precision, accuracy, recall, and F1 score metrics.
- Introduction of a hybrid deep learning approach enabling more precise and detailed analysis of student activity data. Leveraging deep learning techniques and accelerometer data from smartphones, the system can discern various patterns and trends in student behavior, offering valuable insights into their activity levels and health status. Identification of areas where students are inactive or lacking physical activity empowers educators to implement targeted interventions and support students’ well-being.
- Utilization of accelerometer data for monitoring students’ physical activities, promoting a healthier lifestyle by tracking exercise routines, sedentary behavior, and overall fitness levels. This information facilitates the encouragement of healthier habits, prevention of health issues, and improvement of overall well-being. Additionally, the correlation between physical activity and health with cognitive function and academic performance underscores the importance of monitoring students’ activities. Educators and parents can use this insight to implement strategies that enhance both physical and academic development.
- Leveraging edge computing for real-time processing of vital signals directly on the smartwatch or a nearby edge device, allowing instant analysis without transmitting large amounts of raw data to a central server. This enables timely response and decision-making, particularly advantageous for critical signal analysis scenarios.

The following content provides an overview of the remaining sections. In the second section, we describe the latest research with sensor-based activity detection utilizing both DL and ML approaches. In section “Proposed method”, the empirical dataset is delineated and the proposed model is explained in detail. A comparison is made between the experimental results and the latest cutting-edge methods in “Experimental results and analysis” section. Moreover, in “Experimental results and analysis” section, we examine an ablation study that demonstrates the full range of applications of our models, including activity detection. In “Conclusion”, section the research work has been concluded and completed based on the findings.

## Related work

Traditional machine learning techniques require extensive feature engineering and domain knowledge to gather the most useful and relevant features from raw sensory data. The most commonly employed machine learning techniques for HAR include Naïve Bayesian (NB)^13^, Random Forest (RF)^14^, Gaussian Mixture Model (GMM)^15^, K-Nearest Neighbor (k-NN)^16^, Hidden Markov Model (HMM)^17^, and Support Vector Machine (SVM)^18^. Deep learning algorithms are being used by researchers to address HAR challenges. HAR modeling is primarily based on deep learning (DL), which produces effective feature learning patterns rapidly and reliably. Deep learning algorithms such as LSTMs, RNNs, and CNNs automate the process of detecting features and categorizing data. DL techniques have shown promise in HAR and have also improved results in 2D-human face recognition and content-based image retrieval.

When CNNs were used to extract temporal data from HAR tasks, performance improved significantly^19–23^. An enhanced architecture for CNN was proposed in^24^, which eliminated pooling layers and included strides in convolutional layers. In several instances, the model’s performance was either preserved or enhanced, while the computational time was reduced. CNNs were built with Lego filters by^25^. CNNs are composed of filters of different dimensions. It eliminates the need for a dedicated network. Their CNNs perform better than traditional CNNs and require less computing time and memory than traditional CNNs, as demonstrated by empirical results.

Using multimodal sensors^26^, suggested an HConvRNN architecture for detecting different actions. Employing a hierarchical approach, the suggested architecture integrates RNNs with CNNs. RNN models leverage signal dynamics temporal correlations, whereas CNNs incorporate the intra-sensor interactions of multi-modal sensors to establish inter-sensor associations. To address the research challenges related to achieving high accuracy, managing computational complexity, minimizing implementation costs, and reducing energy consumption, the authors^27^ presented a magnetic induction system. Deep RNNs have been applied to evaluate the recommended system technique’s performance. HARNNs were proposed by the authors^28^ as WiFi CSI-based deep RNNs for HAR using a range of commercial WiFi appliances. Comparing the emprical results with benchmark approaches, it is evident that they perform better in typical interior scenarios. In order to automatically extract effective feature sets from the infrared input, the authors^29,30^ used the LSTM, which is a kind of RNN. According to the findings of the study, routine daily behaviors can be accurately recognized with a precision rate of 98.28%, unlike existing machine-learning techniques, which are costly but have potential as privacy-protection methods. The study^31^ proposed a hybrid LSTM architecture called 4-layer LSTM-CNN to enhance HAR outcome. In comparison to previous state-of-the-art techniques, the recommended 4-layer LSTM-CNN architecture achieves an average accuracy gain of 2.24% in activity identification. As a result of deep learning models’ ability to represent features, the sensor-based HAR community has demonstrated significant potential in using them. These models, however, require substantial resources, which might hinder their widespread adoption^32^.

Using wearable sensors, Luwe et al.^33^ developed a hybrid deep learning model called 1D-CNN-BiLSTM for detecting human activity. This model combines a one-dimensional CNN and bidirectional long- and short-term memory. From sensor time series data, one-dimensional CNNs extrapolate essential patterns. Gate mechanisms encode feature connections over long distances in a BiLSTM system. With 1D-CNN-BiLSTM, recognition rates are 95.48% on the UCI-HAR dataset, 94.17% on Motion Sense, and 100% on Single Accelerometer.

As mentioned earlier, traditional machine learning techniques such as Naïve Bayesian, Random Forest, Gaussian Mixture Model, K-Nearest Neighbor, Hidden Markov Model, and Support Vector Machine have been extensively employed in HAR due to their ability to leverage domain knowledge and perform effective feature engineering. These methods offer the advantage of interpretability and transparency in model outputs. However, they often require manual feature extraction, which can be time-consuming and may not capture the intricate patterns present in raw sensory data. Additionally, traditional machine learning approaches may struggle with handling high-dimensional data and complex temporal dependencies. In contrast, deep learning algorithms including LSTM, RNNs, and CNNs have gained prominence in HAR due to their capability to automatically learn hierarchical representations from raw data. Deep learning models excel in capturing complex patterns and temporal dependencies, leading to improved accuracy and generalization performance. However, they may require substantial computational resources and large amounts of labeled data for training. Moreover, deep learning models are often perceived as “black-box” systems, lacking interpretability and transparency in their decision-making processes. While traditional machine learning techniques offer interpretability and transparency, they may struggle with complex data representations. On the other hand, deep learning algorithms excel in capturing intricate patterns but may require significant computational resources and lack interpretability. Therefore, the choice of approach should be carefully considered based on the specific requirements and constraints of the HAR task at hand.

## Proposed method

**Figure 1:**
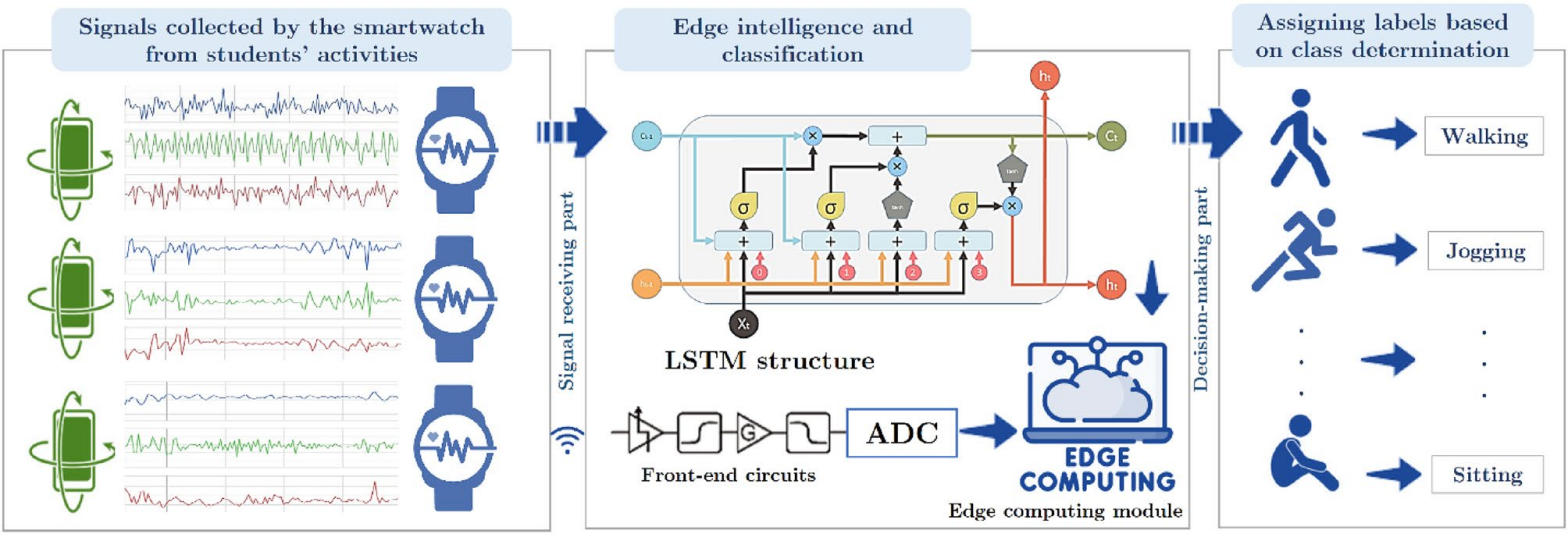
Based on accelerometer data from smartphones with edge computing, this figure illustrates a hybrid deep approach to recognizing student activity and monitoring health physique.

Using accelerometers on their smartphones, we analyzed the activity data of the students to determine their current health status. In Fig. 1, we illustrate the results of our efforts to develop a method for assessing students’ physical fitness levels during different activities. Through smartwatches, the experimental paradigm specifically designed for this purpose successfully recorded the signals of many students. Moreover, our techniques were applied to preprocess the original signals, ensuring their cleanliness for further investigation. Afterwards, we examine the attributes derived from the processed data to discern the behaviors of the students. As a result of the assessment standards, the final evaluation was based on them. An enhanced model assessment framework was used to classify the students’ levels of physical activity. Data processing at the edge reduces transmission latency and bandwidth requirements because it occurs near the source of the data. By analyzing accelerometer data from smartphones in real-time, the hybrid deep approach for student activity detection and health body monitoring enables faster decisions and answers to health concerns. With this system, students’ activities and health can be monitored accurately, with prompt and detailed information provided.

As this study utilized two publicly available datasets, it is essential to provide explanations regarding these datasets. The UCI and WISDM datasets involved the participation of individuals and the utilization of data collected from them. The experiments conducted in this study, utilizing the UCI and WISDM datasets, were validated by the Erasmus Mundus Ph.D. program and the Wireless Sensor Data Mining Lab at Fordham University, respectively. Permissions for using these datasets were obtained in accordance with relevant regulations, and all participants provided informed consent, with parental/legal guardian consent secured for those under 16 years of age.

### Edge computing platform

Edge computing plays a pivotal role in enhancing the analysis of vital signals, especially those obtained from smartwatches, for monitoring the health and well-being of students. For various reasons, we have chosen to incorporate edge computing into the analysis of vital signals from smartwatches in students. Firstly, addressing the challenge of real-time processing, edge computing facilitates instantaneous processing of vital signals directly on the smartwatch or on a nearby edge device. This enables real-time analysis of vital signals without the need to transfer large amounts of raw data to a central server, ensuring timely responsiveness and decision-making^34^.

On the other hand, reducing latency in processing information received from smartwatches for monitoring students’ health is a critical concern. Edge-based analysis of vital signals minimizes latency, as data does not need to be transferred to a distant cloud server for processing. This is particularly important in healthcare scenarios where swift response times are essential, especially in emergencies or situations requiring immediate attention, ensuring timely decision-making. Furthermore, edge computing contributes to the preservation of privacy and security. Local processing of vital signals at the edge reduces the necessity of transmitting sensitive health data to a centralized cloud. This not only safeguards the privacy of students’ health information but also alleviates security concerns associated with transmitting sensitive data over networks.

By employing edge computing, we have the flexibility to customize analysis algorithms based on individual preferences or specific health conditions. This enables personalized health monitoring and analysis tailored to each student’s unique needs. Ultimately, scalability is achieved, as edge computing supports distributed processing, allowing multiple edge devices to work in parallel and handle a larger number of students’ vital signals simultaneously.

### BiLSTM

BiLSTM perform better than other recurrent neural networks in capturing sequential data and making accurate predictions. Their unique ability to model both forward and backward dependencies allows for better representation and comprehension of sequential data. This leads to improved model performance on various tasks. Examples of student activities that can be accurately recognized using BLISTM architecture include walking, running, climbing stairs, sitting still for extended periods, and even activities like yoga or dancing. This level of accuracy allows for personalized insights into students’ physical health and activity levels, helping educators tailor interventions and support accordingly. There may be technical obstacles such as ensuring the accuracy and reliability of data obtained from the smartwatches, as well as integrating the data into existing systems for analysis and intervention. In this study, the LSTM network incorporates three “gates”—the input gate (*i*_*t*_), the forget gate (*f*_*t*_), and the output gate (*o*_*t*_)—to effectively control data flow. In order to effectively handle the deletion of a large volume of data at the last moment, we can employ the forget gate, denoted as *f*_*t*_:

1 $$f_{t} = \sigma \left( {W_{f} \cdot \left[ {h_{t - 1} ,x_{t} } \right] + b_{f} } \right)$$

The input gate or *i*_*t*_ regulates the quantity of data that requires immediate storage.

2 $$f_{t} = \sigma \left( {W_{i} \cdot \left[ {h_{t - 1} ,x_{t} } \right] + b_{i} } \right)$$

The current number of neurons that need to be sent to the next neuron is regulated by the output gate, *o*_*t*_.

3 $$o_{t} = \sigma \left( {W_{o} \cdot \left[ {h_{t - 1} ,x_{t} } \right] + b_{c} } \right)$$

4 $$\tilde{C}_{t} = \tanh \left( {W_{o} \cdot \left[ {h_{t - 1} ,x_{t} } \right] } \right) + b_{c}$$

5 $$C_{t} = \left( {f_{t} *C_{t - 1} } \right) + \left( {i_{t} *\tilde{C}} \right)$$

6 $$h_{t} = \left( {o_{t} *tanh\left( {C_{t} } \right)} \right)$$

**Figure 2:**
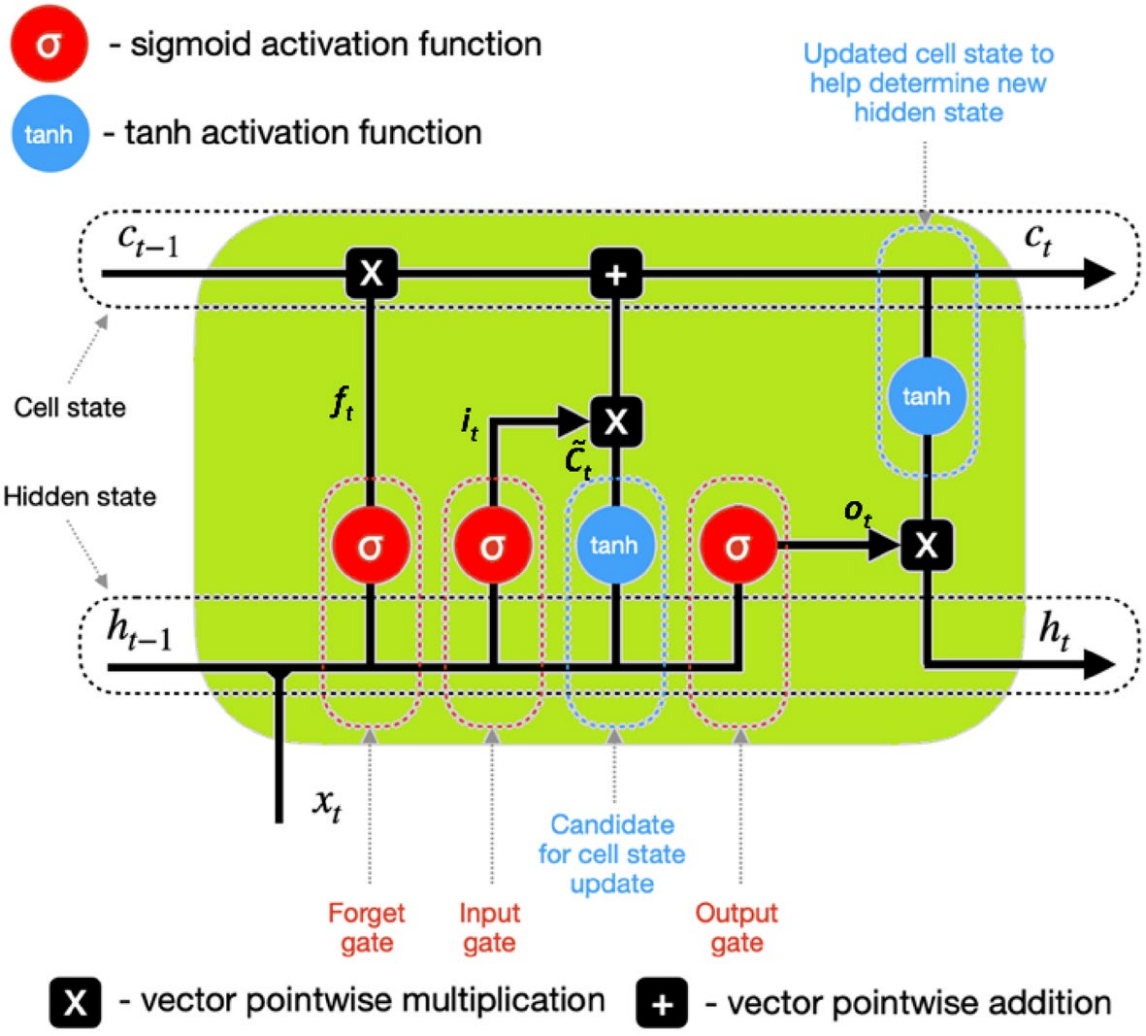
An overview of the construction of the LSTM module.

The weight matrix is denoted as *W*_(*⋅*)_, *h*_*t−*1_ represents the previous output of the network, *h*_*t*_ represents the current final output of the network, and *C*_*t*_ represents the internal variable of the LSTM network that stores data up to the current instant. At this same time, the candidate state is denoted by the letter *C̃*_*t*_. The input is represented by *x*_*t*_, the bias term is denoted as *b*_(*⋅*)_, the logistic function is represented by *σ*_(*⋅*)_, and the activation function is tanh_(⋅)_. The cyclic unit structure of the LSTM cell is seen in Fig. 2.

7 $$\sigma \left( x \right) = \left( {1 + exp\left( { - x} \right)} \right)^{ - 1}$$

8 $$tanh(x) = 2\sigma \left( {2x} \right) - 1$$

At time *t*, a LSTM cell receives three inputs: the previous time’s result (*h*_*t−*1_), the current state of the cell (*C*_*t−*1_), and the value of the network input (*x*_*t*_). The cell can perform its functions with the assistance of these three inputs. At any given moment, the cell produces two outputs: the *h*_*t*_ output value and the current cell state, denoted as *C*_*t*_.

**Figure 3:**
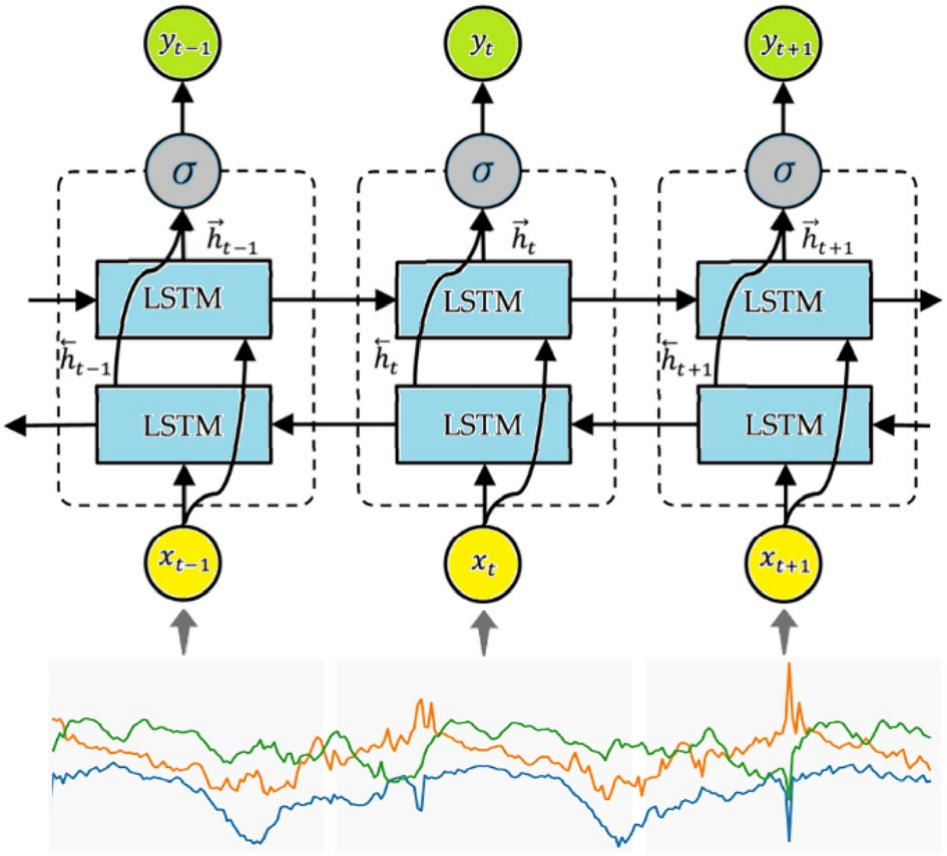
This illustration depicts the architecture of the BiLSTM network used for recognizing student activity based on signals received from a smartwatch.

There are actions that rely on both preceding and subsequent information to determine the outcome at a given point. Therefore, in these problems, an additional network layer is incorporated and sent in reverse chronological order to enhance the network’s learning capacity. The BiLSTM consists of two LSTM network layers that share the same inputs but exchange data in opposing ways. An illustration of the BiLSTM network used for recognizing student activity is shown in Fig. 3. The layers in this model are: input layer, BiLSTM layer, dropout layer, fully connected layer, softmax layer, and classification layer. To mitigate overfitting, the dropout layer is incorporated.

### Bayesian learning-BILSTM

Implementing Bayesian optimization as an optimization approach greatly simplified the process of tuning hyperparameters and optimizing various problems^25,26^. Using the tested combinations, it forecasts the next viable hyperparameter combination that yields the most benefit. The probability density function *p*(*f*(*x*)*|x*) for a hyperparameter optimization function *f(x)* assumes a normal distribution, given that a Gaussian distribution is assumed. Bayesian optimization uses Gaussian process regression to model the present *N* sets of test outcomes, denoted $H = \left\{ {x_{n} ,y_{n} } \right\}_{n = 1}^{N}$. Next, it calculates the posterior distribution of *p*(*f*(*x*)*|x,H*) for the function *f*(*x*) and takes *y*_*n*_ as the observed value of *f*(*x*_*n*_). This process is repeated until the ideal hyperparameters for the LSTM neural network are discovered. As shown in Algorithm 1, a step-by-step procedure is provided for applying Bayesian optimization.

Both the initial learning rate and the number of LSTM cells directly impact the network’s categorization performance. Through Bayesian optimization, the BiLSTM network is capable of finding its ideal values for the initial learning rate and the number of hidden cells in the LSTM layer. Bayesian optimization often utilizes the Expected Improvement function (EI_f_), also known as (9), as its objective function. Adjusting the model’s hyperparameters can further enhance performance.

9 $$EI_{f} \left( {x,H} \right) = \mathop \smallint \limits_{ - \infty }^{ + \infty } max(y^{*} - y,0)p\left( {\left. y \right|x,H} \right)dy$$

Bayesian optimization is a probabilistic model-based optimization technique that can be used to efficiently optimize the architecture of complex machine learning models, such as BiLSTM networks.


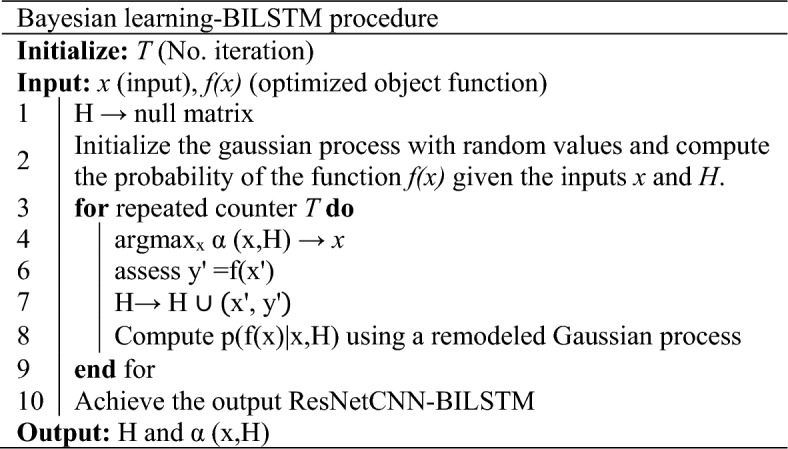


#### Algorithm 1.

The steps in the Bayesian algorithm process used to optimize the BiLSTM architecture.

The process involves iteratively selecting a set of hyperparameters, evaluating the model’s performance, updating a probabilistic model of the objective function, and then selecting the next set of hyperparameters to evaluate. Based on Table 1, we can use Bayesian optimization to optimize BiLSTM.

**Table 1:** Using Bayesian optimization, BiLSTM is optimized using the steps listed in this table.

(1) Define Hyperparameter Space
Identify the hyperparameters that define the architecture of the BiLSTM network. These may include the number of hidden units, learning rate, dropout rates, and any other relevant architectural choices
(2) Define the Objective Function
Specify the objective function that you want to optimize. In the context of optimizing BiLSTM architecture, this would typically be a performance metric such as accuracy, precision, recall, or F1 score, measured on a validation dataset
(3) Select an Acquisition Function
Choose an acquisition function that guides the optimization process. Common acquisition functions include Probability of Improvement (PI), Expected Improvement (EI), and Upper Confidence Bound (UCB). The choice depends on the specific optimization goals and trade-offs
(4) Choose a Surrogate Model
Bayesian optimization relies on a surrogate model to approximate the objective function. Gaussian Processes (GP) are commonly used as surrogate models due to their flexibility and ability to model uncertainty in the objective function
(5) Initialization
Begin the optimization process by evaluating the objective function with a small number of initial random or manually chosen hyperparameter configurations. This creates the initial dataset for the surrogate model
(6) Iterative Optimization
Iteratively perform the following steps:
Fit Surrogate Model:
Update the surrogate model using the available data (hyperparameters and corresponding objective values)
Select Next Hyperparameters:
Use the acquisition function to suggest the next set of hyperparameters to evaluate. This balances exploration and exploitation, aiming to find promising regions of the hyperparameter space
Evaluate Objective Function:
Evaluate the objective function with the selected hyperparameters on the real system (train and validate the BiLSTM with the given architecture)
Update Dataset:
Add the new data point (hyperparameters and objective value) to the dataset
Repeat:
Repeat the process until a stopping criterion is met, such as reaching a specified number of iterations or achieving satisfactory performance
(7) Exploitation-Exploration Balance
Adjust the acquisition function parameters to control the balance between exploitation (focusing on known good regions) and exploration (exploring uncertain regions). This helps fine-tune the optimization process
(8) Final Model Selection
Once the optimization process is complete, select the hyperparameters that correspond to the best-performing model according to the objective function

Applying Bayesian optimization in this manner helps efficiently search the hyperparameter space and find an optimal or near-optimal BiLSTM architecture for a given task. It is particularly useful when the architecture’s performance depends on a complex interplay of hyperparameters, and an exhaustive search is computationally expensive or impractical.

### Ethical approval for animals and human participants

Not applicable (As a disclaimer, the authors are neutral about the ethical issues regarding the dataset preparation, and declare that the standard and common datasets from the other resources have been used. Thus, no new consent and ethical approval action are needed). The study involved the participation of students and utilized data collected from them. For the UCI and WISDM datasets, all experiments conducted in this study were approved by the “Erasmus Mundus Joint Doctorate in Interactive and Cognitive Environments, which is funded by the EACEA Agency of the European Commission under EMJD ICE FPA n 2010–0012” and the “WIreless Sensor Data Mining (WISDM) Laboratory, Department of Computer & Information Science, Fordham University, Bronx, NY,” respectively. These approvals were obtained in accordance with relevant guidelines and regulations. Informed consent was obtained from all participants prior to their involvement in the study. For participants under the age of 16, consent was obtained from their parent and/or legal guardian.

## Experimental results and analysis

### Datasets

A total of thirty individuals provided their activity levels using their Samsung Galaxy S II smartphones for the UCI HAR dataset^35^. More specifically, it encompasses signals from a gyroscope with a frequency of 50 Hz and signals from a linear acceleration sensor on three axes. Table 2 provided in this document defines the sample number of the UCI Human Activity Recognition (HAR) dataset, specifically for acceleration signals. After noise filtering as a preliminary step, sensor signals are collected by sampling them with a fixed-width sliding window of 2.56 s, with a 50% overlap. The Butterworth low-pass filter is employed to separate the acceleration data obtained from the sensors into two distinct components: body acceleration and gravity acceleration. A filter with a cutoff frequency of 0.3 Hz was employed since gravitational force is mostly composed of low-frequency elements.

**Table 2:** The table provided in this document defines the sample number of the UCI Human Activity Recognition (HAR) dataset, specifically for acceleration signals.

Type of activity	No. training samples	No. testing samples
Walking downstairs	986	420
Walking upstairs	1073	471
Walking	1226	496
Lying	1407	537
Standing	1374	532
Sitting	1286	491
Total	7352	2947

Each window was used to construct a feature vector by calculating variables in both the time and frequency domains. 561 properties are extracted from each frame using factors in both the time and frequency domains. To obtain a comprehensive account of 561 attributes, one might refer to^35^. The dataset consists of 7352 training records and 2947 test records, totaling 10,299 records in total. In terms of the second dataset, it is referred to as the WISDM dataset^36^. The dataset consists of gyroscope and 3-axial acceleration signals obtained from 51 people using Samsung Galaxy S5 smartphones. All participants completed the eighteen activities listed in Table 3. The mean duration for collecting 54 min of activity data (18 × 3 min) per person was 70 min. The gyroscope and acceleration data were captured at 20 Hz. Each segment consists of 200 readings, taken at 20 readings per second for 10 s. From the raw time series data of each sensor, 92 high-level features are generated for each segment. The segments are non-overlapping and last 10 s. A total of 16,151 acceleration signal samples were utilized for training, while 6923 samples were employed for testing.

**Table 3:** The table in this document outlines the sample numbers for the WISDM Activity Recognition dataset.

Type of activity	Abbreviation	No. samples
Fold clothes	F_Clt	1261
Clapping	Clp	1270
Writing	Wrt	1241
Dribbling	Drib	1413
Catch	Ctc	1431
Kicking	Kik	1466
Drinking	Drk	1226
Eat pasta	E_pas	1407
Eat chips	E_cip	1374
Eat soup	E_sup	1286
Eat sandwich	E_san	1242
Brush Teeth	Br_Tth	7352
Typing downstairs	Dwn_Str	1180
Standing upstairs	Stn_upstr	1283
Sitting	Sit	1263
Stairs	Str	1180
Jogging	Jagg	1314
Walking	Wlk	1271
Sum total	–	23,074

### System and model setting

We utilized the recommended classifier to assess the model’s capability to handle diverse states. The data was split into two segments: training and testing. Employing k-fold cross-validation (CV), specifically fivefold CV stratified by subject (student), enabled us to evaluate the algorithm’s performance and obtain validation data. To achieve optimal convergence for all convolutional hybrid architectures, it was imperative to minimize training and validation errors. If accuracy tests consistently failed or mistake rates did not decrease, training was halted immediately. Analyzing the accuracy and computing time of four sets of smartphone accelerometer data using different models revealed that the BiLSTM- Bayesian optimization model exhibited the most efficient computational rate, the fewest parameters, and yielded the most favorable experimental outcomes. Consequently, our model excels in predicting real-time load signals from smartphone accelerometers in the smart grid.

### Evaluations

An approach to assess a model involves examining its accuracy, sensitivity, and specificity, which collectively provide a measure of the overall number of accurate predictions it generates. The Eqs. (10)–(12) pertain to accuracy and are expressed as follows:

10 $$Specificity = \left( {\frac{{N_{TN} + N_{TP} }}{{N_{TN} + N_{TP} + N_{FP} + N_{FN} }}} \right)$$

11 $$Specificity = \left( {\frac{{N_{TN} }}{{N_{TN} + N_{FP} }}} \right)$$

12 $${\text{Sensitivity}} = \left( {\frac{{N_{TP} }}{{N_{TP} + N_{FN} }}} \right)$$

Table 4 presents a juxtaposition of the test results acquired using our methodologies. The results for the WISDM dataset are also displayed in Table 5, showcasing the evaluation metrics for each specific class. This comprehensive breakdown allows for a detailed examination of the model’s performance across various categories. Moreover, by isolating the evaluation values for each class, we gain valuable insights into the model’s precision, recall, and F1-score tailored to distinct aspects of the dataset. In the context of the initial dataset (UCI), an observation reveals that, on average, the accuracy for the “Walking downstairs” and “Walking upstairs” classes is comparatively lower than for other classes.

**Table 4:** Estimating the classification accuracy based on the proposed method, categorizing classes in the UCI dataset.

Classes	Method	Average of five-folds				
		Precision	Sensitivity	Specificity	Accuracy	F1-measure
Walking downstairs	LSTM	82.10	93.21	95.61	95.03	88.56
	biLSTM	83.20	94.25	96.05	95.74	89.16
	LSTM + Baysian learning	85.44	95.14	96.84	96.12	90.08
	biLSTM + Baysian learning	87.98	96.06	97.02	96.41	91.84
Walking upstairs	LSTM	93.11	83.96	97.45	94.88	88.99
	biLSTM	93.93	84.89	98.10	95.22	89.80
	LSTM + Baysian learning	94.75	85.02	98.56	95.89	90.01
	biLSTM + Baysian learning	95.02	85.92	99.06	96.41	90.24
Walking	LSTM	96.63	96.59	94.79	94.72	92.23
	biLSTM	97.56	97.23	94.85	95.55	94.58
	LSTM + Baysian learning	98.81	98.60	96.16	96.28	97.25
	biLSTM + Baysian learning	1.00	99.41	1.00	99.74	99.87
Lying	LSTM	95.58	94.61	97.84	95.08	94.63
	biLSTM	96.05	95.03	98.19	95.91	96.36
	LSTM + Baysian learning	96.86	96.11	98.75	96.44	98.70
	biLSTM + Baysian learning	97.33	1.00	99.31	99.43	99.02
Standing	LSTM	94.78	93.87	98.06	94.50	94.32
	biLSTM	95.69	94.58	98.84	95.14	95.81
	LSTM + Baysian learning	97.25	95.69	99.25	96.01	96.48
	biLSTM + Baysian learning	1.00	97.87	1.00	96.41	98.92
Sitting	LSTM	94.43	94.12	97.29	96.66	94.60
	biLSTM	95.12	95.30	98.45	97.09	95.14
	LSTM + Baysian learning	97.20	96.28	99.13	97.39	98.59
	biLSTM + Baysian learning	1.00	98.35	1.00	98.41	99.68

**Table 5:** Estimating the classification accuracy based on the proposed method, categorizing classes in the WISDM Activity Recognition dataset.

Classes	Method	Average of 5-folds				
		Precision	Sensitivity	Specificity	Accuracy	F1-measure
Fold clothes	LSTM	88.65	93.28	96.72	94.56	90.47
	biLSTM	89.17	94.04	97.39	96.45	92.29
	LSTM + Baysian learning	91.02	95.32	98.89	98.56	94.22
	biLSTM + Baysian learning	92.86	96.27	1.00	98.89	96.30
Clapping	LSTM	86.30	80.06	94.50	93.01	93.39
	biLSTM	87.69	81.17	95.69	93.40	94.10
	LSTM + Baysian learning	88.45	82.60	97.56	94.13	94.72
	biLSTM + Baysian learning	90.91	83.33	99.65	96.27	96.00
Writing	LSTM	89.36	92.58	95.77	94.90	90.41
	biLSTM	89.90	93.09	97.20	95.41	92.18
	LSTM + Baysian learning	90.12	94.86	98.56	96.58	94.32
	biLSTM + Baysian learning	1.00	96.67	1.00	98.92	97.86
Dribbling	LSTM	80.13	90.02	94.70	92.92	84.12
	biLSTM	82.90	90.79	96.38	93.80	85.45
	LSTM + Baysian learning	84.25	91.10	98.66	94.72	87.16
	biLSTM + Baysian learning	86.67	92.86	99.29	96.27	89.66
Catch	LSTM	94.20	93.20	94.39	93.75	93.74
	biLSTM	95.44	94.13	96.60	94.30	94.28
	LSTM + Baysian learning	97.63	95.11	98.48	95.88	95.17
	biLSTM + Baysian learning	1.00	96.27	1.00	98.75	97.50
Kicking	LSTM	89.22	92.51	95.35	95.14	94.78
	biLSTM	90.85	94.29	96.57	96.45	95.40
	LSTM + Baysian learning	91.69	95.17	98.80	97.39	96.01
	biLSTM + Baysian learning	93.75	96.27	1.00	98.65	97.66
Drinking	LSTM	87.08	78.82	94.62	93.52	80.95
	biLSTM	88.37	79.54	96.47	94.20	82.93
	LSTM + Baysian learning	89.12	80.29	98.20	95.17	84.70
	biLSTM + Baysian learning	90.91	83.33	99.65	96.27	86.96
Brush Teeth	LSTM	89.38	93.02	95.33	94.86	93.71
	biLSTM	90.23	94.58	96.74	95.40	94.05
	LSTM + Baysian learning	92.05	95.69	98.94	96.80	96.13
	biLSTM + Baysian learning	94.81	96.27	1.00	98.20	97.33
Typing downstairs	LSTM	94.91	93.69	95.35	94.72	92.09
	biLSTM	96.50	94.89	96.36	95.20	94.30
	LSTM + Baysian learning	97.25	95.44	98.70	96.12	95.54
	biLSTM + Baysian learning	99.50	96.70	99.51	98.45	97.38
Standing upstairs	LSTM	97.00	93.27	96.32	95.10	94.28
	biLSTM	97.75	94.70	97.52	96.15	95.30
	LSTM + Baysian learning	98.41	95.68	98.11	97.20	96.88
	biLSTM + Baysian learning	99.36	96.54	99.69	98.84	97.44
Sitting	LSTM	96.72	93.60	96.43	96.52	93.26
	biLSTM	97.25	94.81	97.12	97.08	95.01
	LSTM + Baysian learning	98.55	95.44	98.52	97.80	96.29
	biLSTM + Baysian learning	92.31	96.27	1.00	99.65	96.00
Stairs	LSTM	95.48	94.02	96.24	94.07	93.68
	biLSTM	97.13	94.82	98.15	95.49	94.92
	LSTM + Baysian learning	98.70	95.39	98.84	97.55	96.18
	biLSTM + Baysian learning	99.22	96.30	99.70	98.10	97.16
Jogging	LSTM	95.02	93.62	94.24	93.69	91.72
	biLSTM	96.80	94.20	95.69	94.33	94.12
	LSTM + Baysian learning	98.17	95.11	97.36	96.85	96.50
	biLSTM + Baysian learning	99.53	96.74	99.74	98.58	97.89
Walking	LSTM	92.05	92.20	93.77	92.01	90.17
	biLSTM	93.62	94.09	95.41	93.63	92.48
	LSTM + Baysian learning	94.11	95.66	97.66	95.30	94.23
	biLSTM + Baysian learning	96.81	97.27	98.13	97.41	96.20

Furthermore, the disparity between these two classes, when classified against each other, exhibits a higher level of error than when compared to the overall classification performance. The classification accuracy notably excelled when the individual was in the “Lying” state, demonstrating superior and more fitting performance compared to other states. Moreover, the categorization of other classes, based on signals derived from smartphone accelerometers, is also assessed to be satisfactory, affirming the robustness of the model across various activity states. Furthermore, we evaluated the F-measure, commonly referred to as the F1-score, as a metric that amalgamates precision and recall (sensitivity) into a unified value, offering a well-balanced assessment of a model’s efficacy. Similarly, in the second dataset, the computation related to performance measurement metrics was conducted; however, the reporting of certain human actions was omitted. These instances were disregarded, possibly due to limited investigation in educational settings, although the method performed in a manner such that their classification accuracy percentage did not exhibit a high standard deviation.

**Figure 4:**
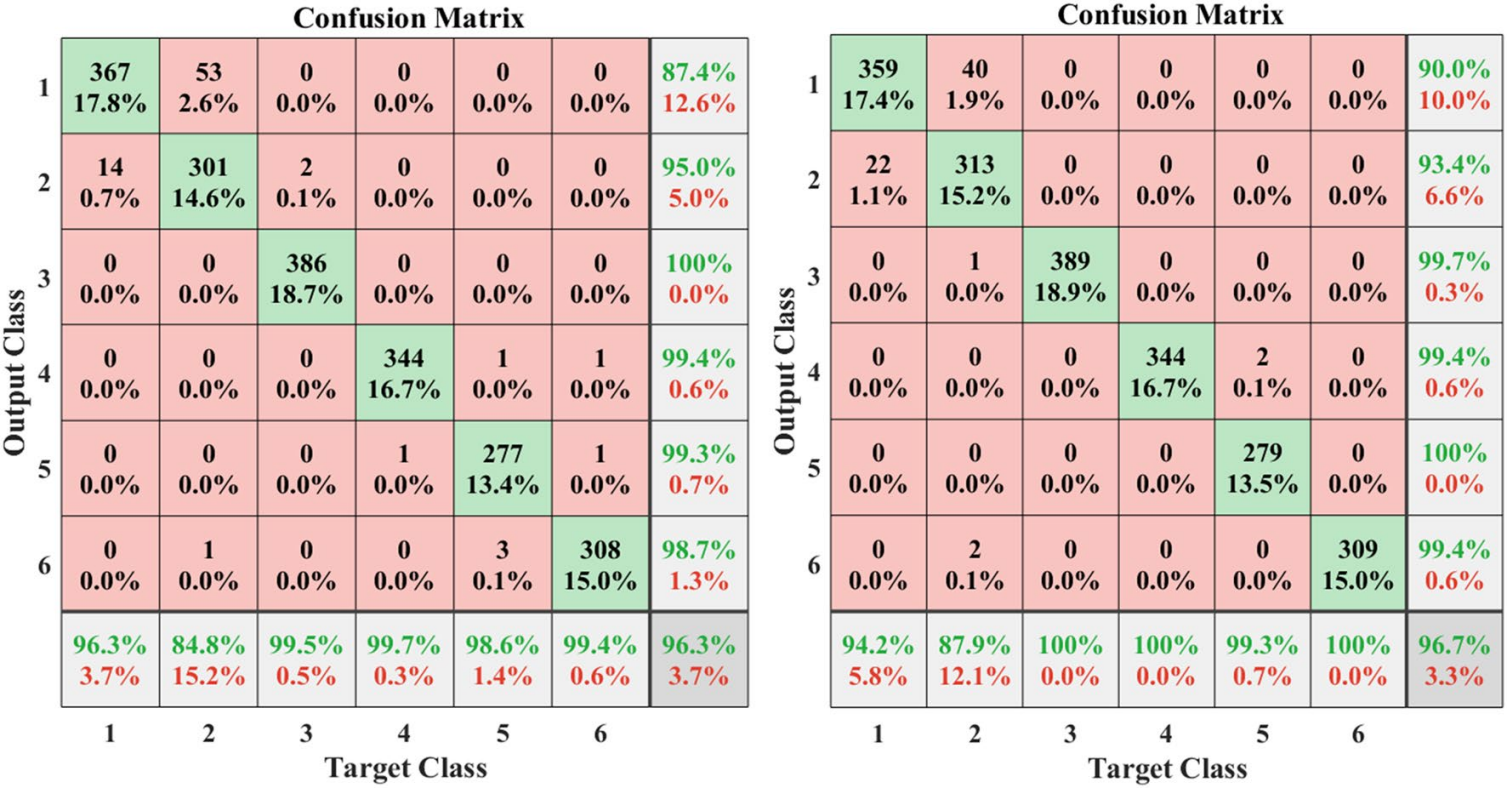
The accuracy of the UCI dataset is illustrated based on the evaluation, presenting the confusion matrix. The classes include Standing (Class 1), Sitting (Class 2), Lying (Class 3), Walking (Class 4), Walking downstairs (Class 5), and walking upstairs (Class 6). Two random samples of these matrices have been presented, revealing that in each instance, the standard deviation is low. This indicates that the proposed method consistently performs well with robust functionality.

**Figure 5:**
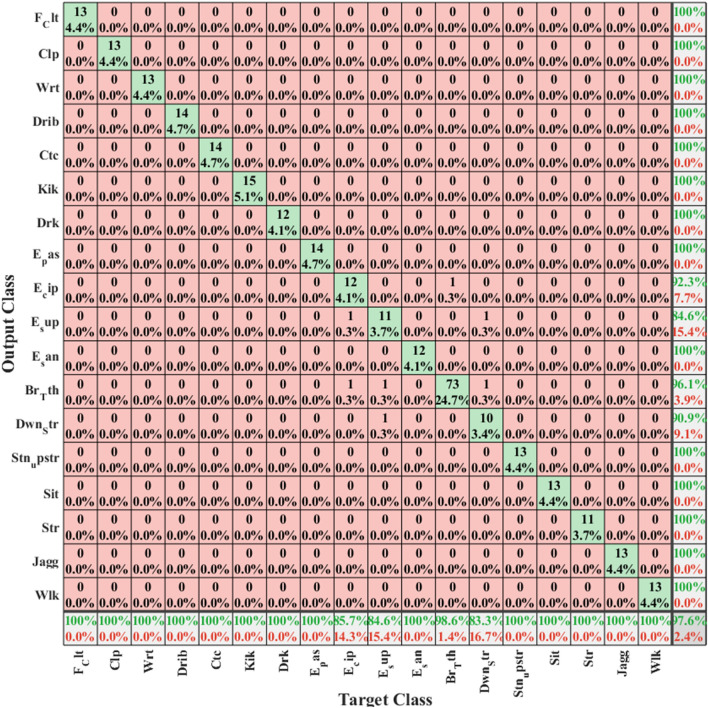
The accuracy of categorizing classes in the WISDM Activity Recognition dataset is illustrated through the evaluation, showcasing the confusion matrix for test signals treated as random data (five-fold 1).

**Figure 6:**
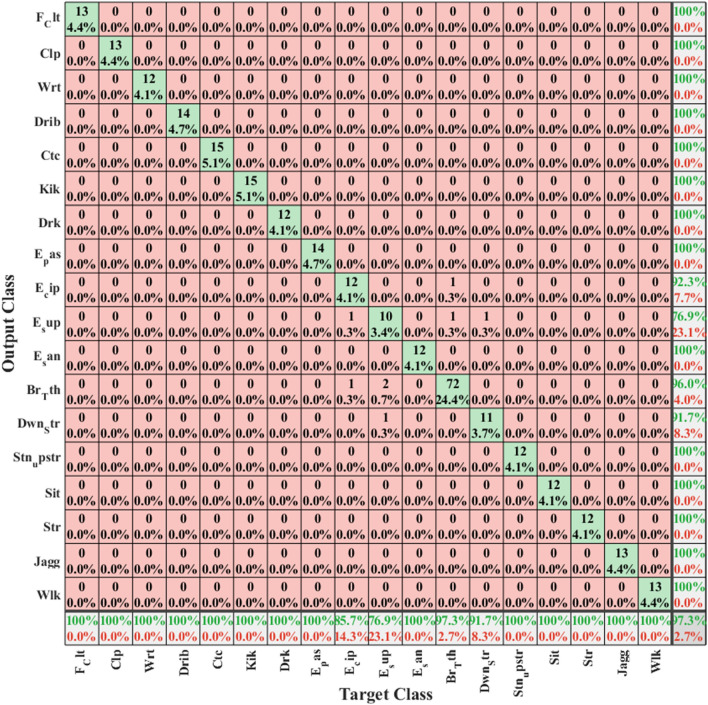
The evaluation reveals the accuracy of class categorization in the WISDM Activity Recognition dataset, presenting the confusion matrix for test signals treated as random data (fivefold 2).

**Figure 7:**
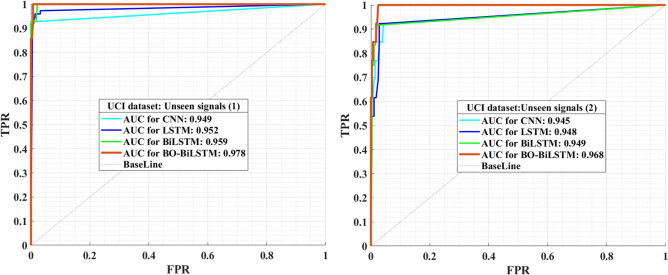
The ROC curves of UCI dataset**.** Based on accelerometer data from smartphones, the ROC curves illustrate the performance of the proposed model (as red color) in recognizing student activity and monitoring health. According to the curves, the AUC value obtained is satisfactory.

Within the classes pertaining to the second dataset, “Sitting,” “Kicking,” and, ultimately, “Catch” exhibited higher accuracy in recognition compared to other classes. However, the accuracy of classification for “Clapping” and “Drinking” classes was comparatively lower than that of the other classes. It’s important to note that Figs. 4, 5, and 6 depict the confusion matrices for all classes of these two datasets, respectively. Since the configuration of the confusion matrix is based on 18 distinct classes, it necessitates running the algorithm twice for different actions. The visual representation of humans utilizing smartphone acceleration signals is highlighted in the second dataset, hence illustrated in two separate figures (refer to Figs. 6 and 7). The showcased random samples of these matrices (See Figs. 5 and 6) not only demonstrate their structure but also highlight that, in each instance, the associated standard deviation is minimal. This low standard deviation is indicative of the proposed method consistently delivering strong and reliable performance. The reliability and stability observed in these matrix samples underscore the efficacy of the proposed approach across diverse scenarios and reinforce its functional strength in handling classification tasks within the evaluated datasets.

### Discussion

**Figure 8:**
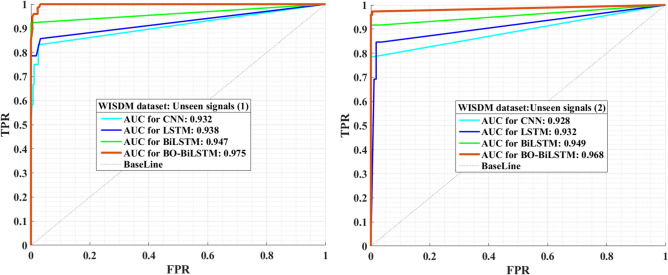
The ROC curves of WISDM dataset. Derived from smartphone accelerometer data, the ROC curves visually present the proposed model’s efficacy (highlighted in red) in recognizing student activity and monitoring health. As indicated by the displayed curves, the achieved AUC value is considered satisfactory.

The research introduces a novel approach by advocating the use of Bayesian optimization techniques to fine-tune the hyper-parameters of the BiLSTM neural network. Possible approaches to validating the accuracy of the Area Under the Curve (AUC) of the ROC curve (Receiver Operating Characteristic curve) as metrics in this context could involve several strategies. One approach is to compare these metrics to established benchmarks or reference standards commonly used for assessing student activity and physical health. By aligning the ROC curve performance with widely accepted measures, consistency and reliability can be ensured. Another validation approach is to conduct independent studies using diverse accelerometer data types. Comparing the AUC metrics derived from ROC curve analysis with expert assessments or physiological measures, such as heart rate monitoring, can provide valuable insights into the effectiveness of the proposed metrics. Additionally, a robust validation strategy may involve the utilization of longitudinal data. Tracking changes in student activity and physical health over an extended period can offer valuable insights into the accuracy and reliability of AUC metrics. This longitudinal analysis helps to assess the consistency of the metrics in capturing variations and trends over time. Comprehensive validation process for AUC metrics could include comparisons with established benchmarks, independent validation studies using diverse data sources, and the analysis of longitudinal data to ensure the accuracy and reliability of the metrics in the context of assessing student activity and physical health. For both datasets, ROC charts were generated and are illustrated in Fig. 7 and 8. These charts depict the acceleration signals acquired from smartphones that are not visible. AUC values were calculated, indicating the effectiveness of the proposed model in the analysis of physical activity and students’ actions.

The information provided states that a deep learning-based method called Bayesian-BiLSTM has been proposed for classifying accelerometer signals to identify various physical activities of students. This method outperforms others like LSTM and CNN, demonstrating higher AUC values and improved performance. Thus, its interpretation is:

*Improved performance with Bayesian-BiLSTM* This result indicates that the Bayesian-BiLSTM method performs better in classifying the physical activities of students than other methods such as LSTM and CNN. In other words, this approach can provide higher accuracy and better discrimination capability in identifying different types of physical activity.

*Higher AUC values* The increase in AUC values signifies an improvement in the model’s ability to differentiate between categories. With higher AUC values, the likelihood of accurately identifying various categories of physical activity increases.

*Preference for Bayesian-BiLSTM as a method* This result may suggest that in specific environments or conditions, such as identifying students’ physical activities, the use of the Bayesian-BiLSTM method takes precedence over other approaches.

One potential challenge associated with employing Bayesian optimization to optimize the BiLSTM in this context is the intricacy and subjectivity inherent in the data. The accelerometer data collected from smartphones constitutes a complex dataset that necessitates meticulous cleaning and preprocessing. Moreover, navigating the subjectivity involved in recognizing student activity and monitoring health physique based on this data poses a challenge, as it requires interpretations and decisions based on vague and subjective criteria.

**Figure 9:**
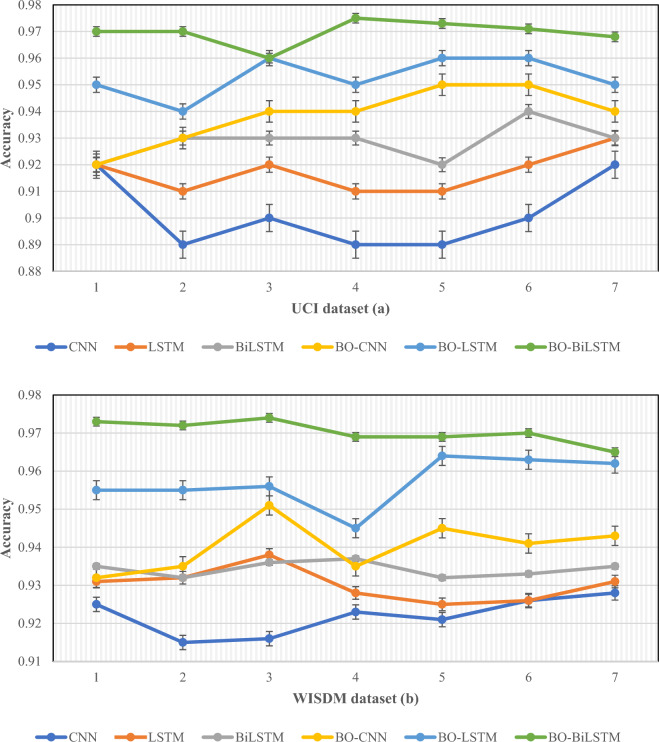
In this figure, the impact of augmenting the outputs in the recognition of students’ actions is depicted by contrasting the results before and after Bayesian optimization (BO) for three models: CNN, LSTM, and BiLSTM, showcased for **a** UCI and **b** WISDM datasets, respectively.

Notwithstanding the intricacies and subjectivity of the data, Bayesian optimization presents several advantages in this scenario. It facilitates the exploration and exploitation of the dataset, enabling the quick identification of promising starting points for optimization and the adaptation of its strategy as needed. Additionally, Bayesian optimization can adeptly handle noisy and incomplete data, rendering it suitable for optimizing the BiLSTM model using accelerometer data from smartphones. Furthermore, its flexibility allows for the incorporation of prior knowledge or domain expertise, potentially enhancing the accuracy and reliability of recognizing student activity and monitoring health physique based on this data. In Fig. 9, the impact of enhancing the outputs in recognizing students’ actions is evident when comparing the results before and after the application of Bayesian optimization.

The Baysian-BiLSTM method, grounded in deep learning, is proposed for the classification of accelerometer signals from student movements to discern various types of physical activities. Its accuracy, when compared to alternative methods including CNN^36–42^, Handcrafted features^41^, LGSRNet^43^, and deep decision fusion^44^, surpasses significantly. In Table 6, we present a comparative analysis of our methodology against other studies utilizing the UCI and WISDM datasets. The results clearly indicate that our proposed approach outperforms the other methods, achieving the highest level of accuracy.

**Table 6:** In this table, a comparative analysis is provided for our methodology in contrast to other studies, utilizing the UCI and WISDM datasets.

Author	Method	Dataset	Metric (accuracy) (%)
Wan et al.^37^	Convloutional neural networks (CNNs)	UCI	93.21
Zheng et al.^38^	Convloutional neural networks (CNNs)	UCI	94.79
Kim et al.^39^	Convloutional neural networks (CNNs)	UCI	95.18
Jiang et al.^40^	Convloutional neural networks (CNNs)	UCI	95.25
Kolosnjaji et al.^36^	Pretrained neural networks + PCA	UCI	96.17
Friday et al.^41^	CNN + Handcrafted features	UCI	96.90
Li et al.^42^	CNN + Enhancing features	UCI	97.12
Zheng et al.^43^	LGSRNet	UCI	97.32
Zhang et al.^44^	Deep decision fusion	UCI	97.81
		WISDM	85.00
Proposed model	BO-BiLSTM	UCI	97.56
		WISDM	97.30

When contrasted with CNN, Baysian-BiLSTM exhibits superior accuracy. While CNN is generally effective for image data, its accuracy may decrease when applied to time-series signals such as those representing student movements. Furthermore, in comparison to the Handcrafted Features method, Baysian-BiLSTM demonstrates heightened accuracy. This underscores the potential of automatic and adaptive learning inherent in deep models, yielding superior outcomes in the analysis of intricate signals. Additionally, when compared to LGSRNet, the Baysian-BiLSTM method achieves heightened accuracy, suggesting the robustness of deep learning models in handling complex and diverse datasets. Moreover, in contrast to the Deep Decision Fusion method, Baysian-BiLSTM attains superior accuracy, possibly indicating its enhanced capability to integrate and make decisions based on deep information. The Baysian-BiLSTM method appears to outperform alternative approaches in identifying various types of physical activities among students, particularly when confronted with time-series and dynamic data.

Edge computing involves performing lightweight computations and inference tasks on edge devices, such as smartphones, edge servers, or IoT devices. These computations are typically less resource-intensive compared to centralized cloud servers, as they involve processing data locally at the network edge. Therefore, the computational complexity of edge computing in our method is relatively low, making it suitable for real-time video analysis and classification on resource-constrained edge devices. Regarding the Bayesian-BiLSTM model, it is essential to consider the computational demands associated with both components:

1. *BiLSTM* BiLSTM has the ability to automatically learn and extract features from raw accelerometer data. This eliminates the need for manual feature engineering, which can be complex and time-consuming.
2. *Bayesian-BiLSTM* The Bayesian-BiLSTM algorithm is considered as an optimization process that takes into account prior information and the entities obtained from the learning process. Using this information, the Bayesian algorithm can provide estimates of the probability distribution of the best parameters for the BiLSTM network. Since the Bayesian algorithm has the ability to estimate the probability distribution of parameter optimization, this method can be executed offline. Thus, implementing the Bayesian-BiLSTM algorithm does not involve any online intervention.

We have outlined several avenues for future research. Firstly, we plan to delve deeper into fine-tuning the hyperparameters of the proposed BiLSTM model. This involves exploring various configurations and settings to optimize its overall performance. Additionally, we aim to implement adaptive strategies for dynamically adjusting model parameters during training. This may include employing techniques such as learning rate schedules or incorporating mechanisms for automatic adjustment based on ongoing model performance. Furthermore, we intend to investigate the potential benefits of ensemble learning techniques. This entails combining multiple BiLSTM models or incorporating diverse model types to enhance classification accuracy and overall robustness.

Our future work will also extend the model to support online learning, enabling adaptation to changes in student activities over time. To achieve this, we will leverage edge computing as a platform for data processing. This approach allows for continuous monitoring and real-time updates based on newly acquired data. In addition, we plan to explore methods for user-specific personalization, tailoring the model to individual behavior patterns of students. This personalized approach is expected to significantly improve the accuracy of activity recognition for each student. In addition to these cases, the use of new deep learning models such as transformer learning^45^, attention models^46^, federated learning^47^ and other new networks can affect the optimal learning process.

Privacy is a paramount concern, especially when dealing with sensitive health-related data. To address this, we will leverage edge computing to explore and implement privacy-preserving techniques. This may involve considering approaches such as federated learning or differential privacy. To provide a more comprehensive understanding of students’ activities and health status, we will incorporate data from additional sensors or modalities, such as gyroscopes and magnetometers. Finally, our research will culminate in real-world deployments of the proposed system, accompanied by the collection of user feedback. This valuable input will guide us in refining the model, enhancing the user experience, and addressing any practical challenges that may arise.

## Conclusion

This research has introduced a robust framework utilizing smartphone sensors for Human Activity Recognition (HAR), addressing the diverse applications and potential benefits in promoting healthier lifestyles among students. By monitoring accelerometer data, valuable insights into students’ physical activities have been gleaned, aiding in the encouragement of healthier habits and the prevention of health issues. Traditionally, HAR relied on handcrafted features for activity analysis, but the integration of deep learning, particularly the LSTM network, has revolutionized this field by enabling automatic feature extraction from raw sensory data, thus enhancing accuracy and scalability. Our proposed method incorporates a feature analysis framework aimed at recognizing student activity and monitoring health physique through accelerometer data from smartphones, leveraging edge computing for efficient processing. Despite challenges such as suboptimal model states due to presetting parameters, we have utilized BiLSTM and Bayesian optimization to improve model performance significantly. Through rigorous validation, our model has demonstrated exceptional classification accuracy, outperforming existing methodologies. Looking ahead, several avenues for future research have been identified. We plan to delve deeper into fine-tuning hyperparameters, exploring adaptive strategies for dynamic parameter adjustment, and investigating the potential benefits of ensemble learning techniques. Moreover, extending the model to support online learning through edge computing, exploring user-specific personalization, incorporating new deep learning models, and addressing privacy concerns with privacy-preserving techniques are areas of focus. Ultimately, this research represents a significant advancement in the field of HAR, with implications for fostering healthier lifestyles among students through innovative technological solutions. Real-world deployments of the proposed system, accompanied by user feedback, will further refine the model and enhance the user experience, driving forward the intersection of technology and health promotion

## Data Availability

All datasets used in this study are freely available through the open repositories on the web. All data generated or analyzed during this study are included in this published articles and its supplementary information files from UCI^35^, http://archive.ics.uci.edu/dataset/240/human+activity+recognition+using+smartphones , and WISDM^36^, http://www.cis.fordham.edu/wisdm/.
